# In Vitro and In Vivo Analysis of Ochratoxin A-Derived Glucuronides and Mercapturic Acids as Biomarkers of Exposure

**DOI:** 10.3390/toxins13080587

**Published:** 2021-08-23

**Authors:** Raphael Dekant, Michael Langer, Maria Lupp, Cynthia Adaku Chilaka, Angela Mally

**Affiliations:** Department of Toxicology, Julius-Maximilians-University of Wuerzburg, Versbacher Strasse 9, 97078 Würzburg, Germany; raphael.dekant@uni-wuerzburg.de (R.D.); michael.langer98@gmx.de (M.L.); maria.lupp@stud-mail.uni-wuerzburg.de (M.L.); adaku80@yahoo.com (C.A.C.)

**Keywords:** ochratoxin A, biomarker of exposure, glucuronide, mercapturic acid, mycotoxin

## Abstract

Ochratoxin A (OTA) is a widespread food contaminant, with exposure estimated to range from 0.64 to 17.79 ng/kg body weight (bw) for average consumers and from 2.40 to 51.69 ng/kg bw per day for high consumers. Current exposure estimates are, however, associated with considerable uncertainty. While biomarker-based approaches may contribute to improved exposure assessment, there is yet insufficient data on urinary metabolites of OTA and their relation to external dose to allow reliable estimates of daily intake. This study was designed to assess potential species differences in phase II biotransformation in vitro and to establish a correlation between urinary OTA-derived glucuronides and mercapturic acids and external exposure in rats in vivo. In vitro analyses of OTA metabolism using the liver S9 of rats, humans, rabbits and minipigs confirmed formation of an OTA glucuronide but provided no evidence for the formation of OTA-derived mercapturic acids to support their use as biomarkers. Similarly, OTA-derived mercapturic acids were not detected in urine of rats repeatedly dosed with OTA, while indirect analysis using enzymatic hydrolysis of the urine samples prior to LC–MS/MS established a linear relationship between urinary glucuronide excretion and OTA exposure. These results support OTA-derived glucuronides but not mercapturic acids as metabolites suitable for biomonitoring.

## 1. Introduction

With the global mycotoxin prevalence in food crops estimated to be as high as 60–80% [1], mycotoxins present an unavoidable, ongoing global threat to food safety and food security. The mycotoxin ochratoxin A (OTA), a potent nephrotoxin and renal carcinogen, is found as a contaminant in a wide variety of foods and is therefore considered a health concern. Besides cereal products, coffee, cocoa, beer, wine and grape juice, which have long been recognized as major food categories contaminated with OTA [2,3], occurrence data submitted to the European Food Safety Authority (EFSA) between 2009 and 2018 combined with European food consumption data also identified fresh and dried fruits, preserved meat, ripened cheese, non-chocolate confectionary and breast milk as important contributors to chronic dietary exposure to OTA [4]. Based on these data, mean chronic exposures for the European population ranging from 0.64 to 17.79 ng/kg body weight (bw) per day (minimum lower bound (LB) to maximum upper bound (UB)) were estimated across dietary surveys and age groups [4]. For highly exposed consumers (95th percentile exposure), chronic dietary exposure estimates range from 2.40 to 51.69 ng/kg bw per day (minimum LB to maximum UB) [4]. The current exposure assessment thus suggests somewhat higher dietary intake of OTA compared to previous estimates that indicated exposure ranging from 2 to 3 ng/kg bw per day for average consumers and 6 to 8 ng/kg bw per day for high consumers [3]. However, EFSA considered that the assessment was associated with a number of uncertainties, including limited consumption surveys and extrapolation of occurrence data provided mainly by two countries and a single study on breastfed infants to the whole of Europe [4].

Biomarker-based approaches are increasingly recognized as an efficient and cost-effective complementary approach to assess human exposure to food contaminants, which may help not only to identify vulnerable and highly exposed consumer groups but also to reduce uncertainties related to occurrence and consumption rates. OTA is frequently analyzed in blood, urine or breast milk to monitor internal human exposure to OTA [4,5]. Due to its long half-life in plasma, concentrations in serum or plasma are considered to reflect intake over a longer period of time, whereas urinary excretion of OTA may be used to monitor acute exposure to OTA. However, relating concentrations of OTA in body fluids to external daily intake is hampered by the long OTA plasma half-life, low urinary excretion rates (e.g., only 2.1 and 5.2% of an orally administered dose of 0.5 mg/kg bw is excreted in the urine of male and female rats, respectively, within 96 h), and limited data on OTA toxicokinetics in humans [4,6,7].

**Figure 1:**
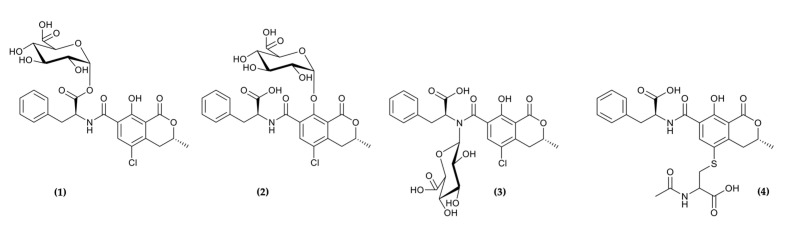
Structures of Ochratoxin A (OTA)-derived glucuronides proposed by [10] (acyl-glucuronide (**1**), phenol-glucuronide (**2**) and amino-glucuronide (**3**)) and structure of OTB-NAC (**4**) reported to be present in human urine.

Besides OTA itself, a number of OTA metabolites have been suggested as biomarkers of OTA exposure. These include the major metabolite OTalpha, which is formed by cleavage of the amide bond between phenylalanine and the dihydroisocoumarine moiety, as well as phase II conjugates of OTA and OTalpha. In particular, indirect liquid chromatography–mass spectrometry (LC–MS) methods analyzing urinary OTA and OTalpha following enzymatic hydrolysis of human urine samples suggest that, at least in humans, a considerable fraction of the dose is excreted as glucuronides of OTA (Figure 1) and OTalpha [8]. In contrast, glucuronic acid conjugates of OTA and OTalpha remain undetected in urine of human and experimental animals by direct MS approaches, possibly because of their low ionization efficiency [8]. Thus, there is currently insufficient knowledge regarding potential species differences in the extent of OTA and OTalpha glucuronide formation and correlation between the urinary excretion of OTA/OTalpha-derived glucuronides and external dose as a prerequisite for estimating dietary intake. To further validate OTA-derived glucuronides as potential biomarkers of OTA exposure, we used an indirect LC–MS method to determine 24 h excretion of OTA-derived glucuronides in urine of rats dosed with OTA at 0, 21, 70 and 210 µg/kg bw for up to 90 days and established a correlation between biomarker levels and external dose. Moreover, in vitro incubations of subcellular fractions (hepatic S9 mix) were used to obtain direct evidence for the formation of OTA-derived glucuronides, including potential species differences in the extent of glucuronidation.

In addition to glucuronic acid conjugates, Sueck et al. (2019) recently reported detection of a mercapturic acid (*N*-acetyl-cysteine; NAC) conjugate of OTB (OTB-NAC) (Figure 1) in human urine samples [9]. Concentrations of OTB-NAC comparable to those of OTA were observed in some urine samples, although overall no correlation between the levels of OTB-NAC and OTA was evident. This putative metabolite, in which the chlorine atom of OTA is replaced by *N*-acetyl-cysteine, was suggested to be formed either via the formation of a reactive quinone or by direct catalysis by glutathione-S-transferases [9]. Since this metabolite was not previously detected in in vitro or in vivo biotransformation studies on OTA, we used urine samples obtained from rats repeatedly administered OTA (up to 2 mg/kg bw) as well as liver S9 fractions from rats, rabbits, minipigs and humans to confirm formation of OTB-NAC and/or its corresponding glutathione (GSH) derivative and to assess potential species differences.

## 2. Results

### 2.1. Analysis of OTA-Derived Glucuronides in Urine of Rats Exposed to OTA and in Subcellular Fractions

**Figure 2:**
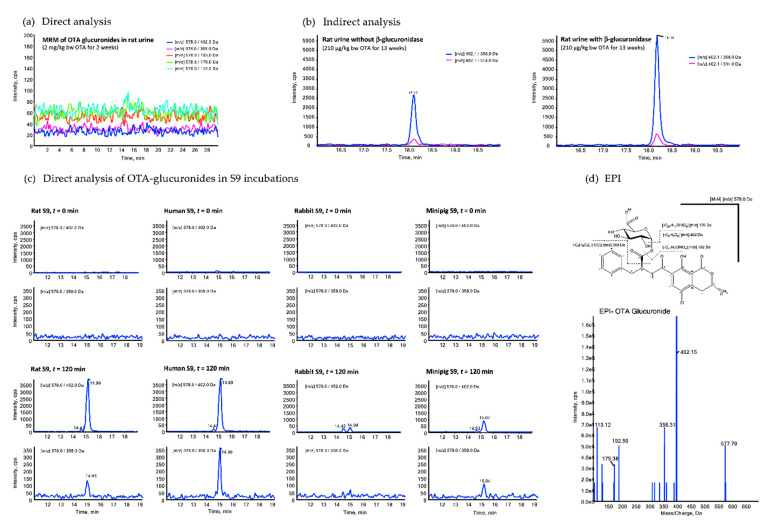
Representative LC–MS chromatograms of urine obtained from rats treated with OTA at 2 mg/kg bw for 2 weeks (**a**) and 210 µg/kg bw for 13 weeks (**b**) obtained by direct (**a**) and indirect analysis with and without β-glucuronidase treatment (**b**). Multiple reaction monitoring of *m/z*-transitions 578/402 and 578/358 demonstrating formation of an OTA-glucuronide in in vitro incubations of OTA with liver S9 from rats, rabbits, minipigs and humans (**c**). Enhanced product ion spectrum [M-H]^−^ showing typical mass fragments of *m/z* 402, 358, 193, 175 and 113, consistent with a previously reported OTA-acyl-glucuronide [10] (**d**).

To date, evidence for the formation of conjugates of OTA with glucuronic acid in vivo comes from indirect analyses of human urine following enzymatic hydrolysis [8]. In order to confirm OTA glucuronide formation and to determine the relationship between external dose and the urinary excretion of OTA-derived glucuronides, we utilized urine samples from repeated dose toxicity studies in rats. Despite the much higher exposure of the experimental animals to OTA (up to 2 mg/kg bw per day) as compared to human dietary exposure, attempts to directly identify and quantify OTA-derived glucuronides by LC–MS/MS using both constant neutral loss (CNL) [(+) 176 Da] and MRM (Figure 2a) failed, presumably due to the reported low ionization efficiency [8]. However, a clear increase in the concentration of OTA was observed in rat urine following enzymatic hydrolysis by β-glucuronidase, providing indirect evidence for OTA-derived glucuronides in rat urine (Figure 2b). In vitro, *m/z*-transitions corresponding to OTA-derived glucuronides (578/402 and 578/358) were detected in incubations of OTA with liver S9, with signal intensities increasing in the order of rabbit < rat/minipig < human (Figure 2c). Enhanced product ion spectra [M-H]^−^ obtained from these signals (Figure 2d) showed typical mass fragments of *m/z* 402, 358, 193, 175 and 113, consistent with the structure of OTA-derived glucuronides and the fragmentation pattern previously assigned to the OTA-acyl-glucuronide [10].

**Figure 3:**
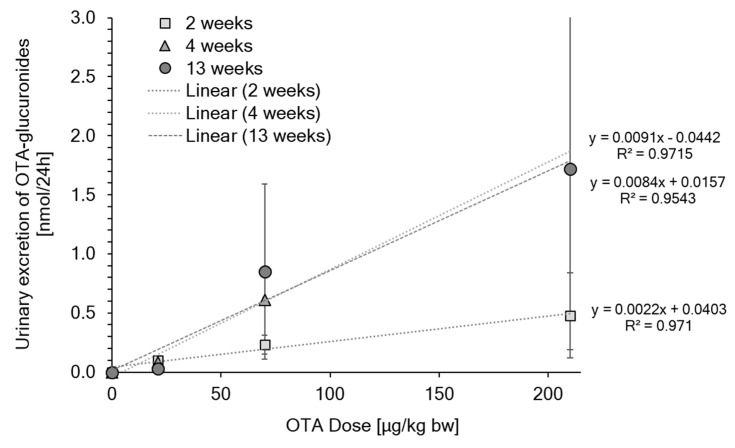
Urinary excretion of OTA-glucuronides within 24 h in rats treated with OTA at 0, 21, 70 or 210 μg/kg per bw (5 days/week) for 2, 4 and 13 weeks. Data are presented as mean ± standard deviation (*n* = 3–5 animals per group). The Pearson correlation coefficients (R^2^) and linear equations for the linear trend lines are displayed.

Quantitative analysis of OTA with and without β-glucuronidase treatment using stable isotope dilution LC–MS demonstrated that in rats, a lower fraction of the administered dose is excreted in the form of glucuronides as compared to the parent compound OTA itself. In rats administered OTA at 2 mg/kg bw for 2 weeks (5 days/week), 24 h urinary excretion of OTA monitored over the course of the study accounted for 0.5–6.9% of the daily administered dose, while indirect analysis of OTA-derived glucuronides suggests that 0.1–2.5% of the daily dose is excreted as glucuronides within 24 h (Table 1). Similarly, in a 90-day study in which rats were dosed with OTA at 0, 21, 70 and 210 µg/kg (5 days/week), the fraction of the administered dose excreted in the form of glucuronides ranged from 0.2 to 1.7% vs. 0.8 to 4.6% for OTA (Table 2). Importantly, the Pearson correlation coefficients demonstrate a linear relationship between OTA dose and urinary excretion of OTA-derived glucuronides across the investigated dose and time (Figure 3).

### 2.2. Analysis of OTB-NAC in Rats In Vivo and in Subcellular Fractions In Vitro

**Figure 4:**
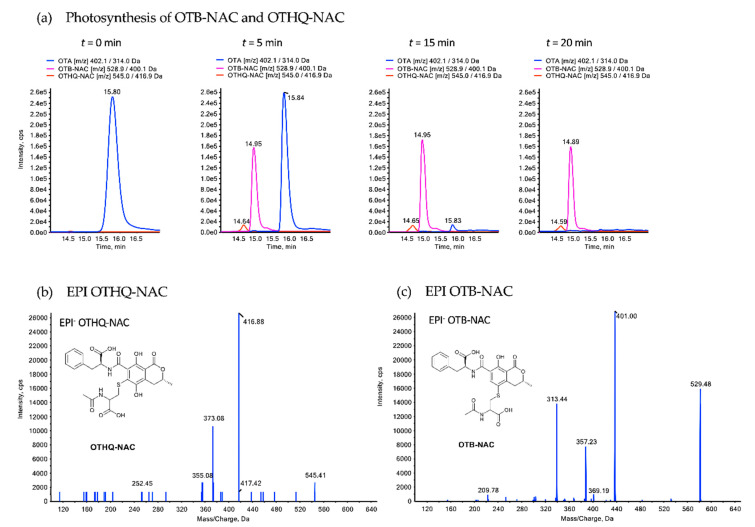
(**a**) LC–MS analysis of the photoreaction of OTA in the presence of NAC, yielding OTB-NAC and OTHQ-NAC. EPI spectra of the NAC conjugates OTHQ-NAC (**b**) and OTB-NAC (**c**).

In order to investigate if biotransformation of OTA in rats in vivo and in subcellular fractions in vitro gives rise to OTB-NAC or its corresponding glutathione conjugate OTB-GSH, OTB-NAC and OTB-GSH were synthesized as reference compounds by photoirradiation of OTA in the presence of *N*-acetyl-cysteine (NAC) and GSH, respectively, and characterized by mass spectrometry (Figure 4). Besides formation of *N*-acetyl-cysteine and glutathione conjugates of OTB, photochemical dechlorination of OTA also gave rise to OTB, OTA-hydroquinone (OTHQ) and its corresponding *N*-acetyl-cysteine (OTHQ-NAC) and glutathione conjugates (OTHQ-GSH), as exemplified in Figure 4 for the *N*-acetyl-cysteine derivatives.

**Figure 5:**
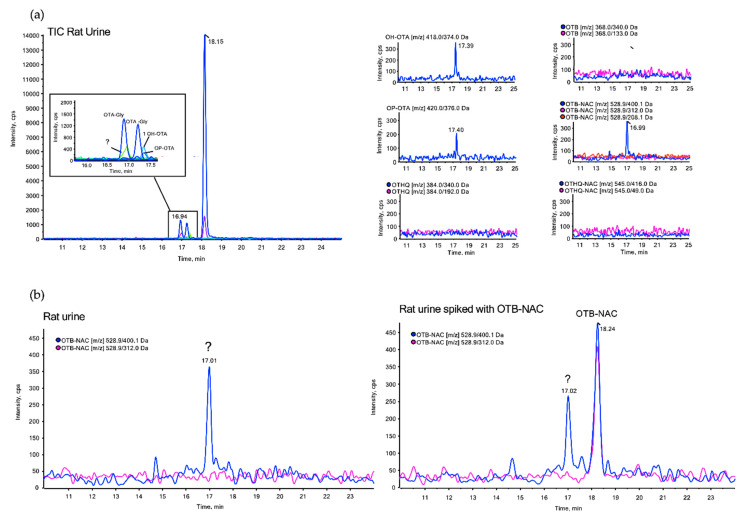
(**a**) LC–MS analysis of urine of a rat treated with OTA at 210 µg/kg bw for 90 days, showing the total ion chromatogram (TIC) and extracted ion chromatograms of *m/z* transitions corresponding to potential OTA-derived metabolites. Besides OTA (RT 18.15), the TIC reveals the presence of the two previously identified glycosides, hydroxy-OTA, traces of OP-OTA and a further signal corresponding to one of the mass transitions of OTB-NAC (*m/z* 529 → 400). (**b**) LC–MS analysis of a spiking experiment in which rat urine was spiked with a solution of OTB-NAC generated by photosynthesis, demonstrating that the unknown compound (*m/z* 529 → 400) present in rat urine independent of OTA treatment is not identical to OTB-NAC.

Since OTB-NAC (but not OTHQ-NAC) was recently reported to be present in urine of human volunteers at similar levels as OTA [9] but has not been previously identified in studies in experimental animals, we speculated that our archived urine samples of rats exposed to OTA at doses several magnitudes above human exposure would allow us to detect this putative OTA metabolite. Urine of OTA-treated rats was analyzed via LC–MS in negative ionization mode, monitoring *m/z* transitions of OTB-NAC (*m/z* 529 → 400/312/208), OTHQ-NAC (*m/z* 545 → 416/49) as well as known metabolites and derivatives of OTA, including hydroxy-OTA (*m/z* 418 → 374), the ring-opened lactone OP-OTA (*m/z* 420 → 376), pentose and hexose conjugates (*m/z* 402 → 358/314), OTalpha (*m/z* 255 → 211/167), OTB (*m/z* 368 → 324/133), 4-hydroxy-OTB (*m/z* 384 → 340) and OTHQ (*m/z* 384 → 340). Besides OTA and OTalpha, hydroxy-OTA, the two glycosides and traces of OP-OTA were consistently found in urine of OTA-treated rats. While OTHQ and OTHQ-NAC were not detected, a signal corresponding to one of the mass transitions of OTB-NAC (*m/z* 529 → 400) was occasionally recorded in urine samples of both untreated controls and animals exposed to OTA. However, this compound did not exhibit further mass fragments characteristic for OTB-NAC (*m/z* 529 → 312/208), which we included in our method as qualifiers. Thus, urine samples in which the signal was detected were spiked with a solution of OTB-NAC generated by photosynthesis. The spiking experiment clearly revealed two separate peaks (Figure 5). This indicates that the compound with mass transition *m/z* 529 → 400 occasionally observed in rat urine independent of OTA exposure does not correspond to OTB-NAC.

**Figure 6:**
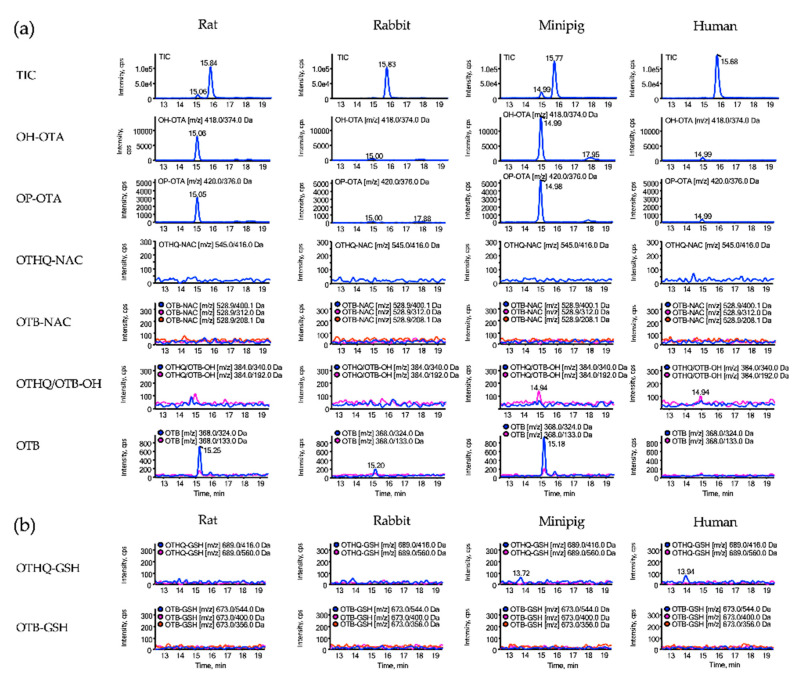
LC–MS analysis of potential OTA metabolites formed in in vitro incubations of OTA with S9 mix of rat, rabbit, minipig and human in the presence of *N*-acetyl-cysteine (**a**) or GSH (**b**).

To understand if species differences in OTA metabolism may give rise to the discrepant findings in humans vs. rats, we used liver S9 from rats, rabbits, minipig and humans in the presence of *N*-acetyl-cysteine or GSH to investigate in vitro biotransformation of OTA to OTB-NAC or OTB-GSH using the same MRM method as described above. In general, time-dependent formation of hydroxy-OTA and OP-OTA was observed in incubations of OTA with rat and minipig liver S9 and, to a lesser extent, in human and rabbit liver S9 (Figure 6 and Supplementary Figure S1–S4). These metabolites were not formed in the absence of the NADPH regenerating system required for cytochrome P450 activity (Supplementary Figure S1–S4). In contrast to hydroxy-OTA and OP-OTA, time-dependent formation of OTA-derived GSH and *N*-acetyl-cysteine conjugates (i.e., OTHQ-GSH, OTHQ-NAC, OTB-GSH, OTB-NAC) was not evident in any of the S9 incubations in the presence of the NADPH regenerating system (Figure 6 and Supplementary Figures S1–S4). However, a signal with a mass transition characteristic for OTHQ-GSH was detected in incubations of rat, rabbit and minipig S9 in the absence of the NADPH regenerating system (Supplementary Figure S1–S4). Although it was not possible to obtain EPI spectra of the metabolite formed, spiking of the incubation mixtures with OTHQ-GSH obtained by photosynthesis revealed identical retention times, suggesting that the metabolite may indeed correspond to OTHQ-GSH.

OTB was detected even at *t* = 0 and no time-dependent increase was evident, indicating that the test compound contained OTB as a minor contaminant. This was confirmed by LC–MS analysis of different batches of OTA used for the in vitro biotransformation studies, which revealed variable low levels of contamination by OTB. time-dependent formation of signals with a mass transition characteristic for both 4-hydroxy-OTB (*m/z* 384 → 340) and OTHQ (*m/z* 384 → 340) was not observed.

## 3. Discussion

In its recent assessment of health risks related to dietary intake of OTA, the European Food Safety Authority (EFSA) identified a need for further studies relating concentrations of potential biomarkers of OTA exposure in body fluids to external dose in order to obtain reliable estimates of daily intakes [4]. Moreover, considering the limited availability of toxicokinetic data on OTA in laboratory animals and humans, EFSA recommended further studies on potential species differences in the toxicokinetics and biotransformation of OTA [4]. To address some of these data gaps, we focused on OTA-derived glucuronides and mercapturic acids as potential biomarkers of OTA exposure to establish a correlation between urinary metabolite levels and external exposure in rats in vivo and to assess potential species differences in phase II metabolite formation in vitro. Overall, our results provide further evidence to support OTA glucuronides as potential biomarkers of exposure, yet do not confirm OTA-derived mercapturic acids as metabolites suitable for biomonitoring approaches.

Our in vitro analyses of OTA biotransformation using liver S9 of rats, humans, rabbits and minipigs clearly confirm formation of an OTA-glucuronide, consistent with a previous study using rat liver microsomes [10]. While OTA may, in principle, form a phenol-, an acyl- or an amino-glucuronide, the EPI spectrum obtained from the OTA-glucuronide formed in our in vitro incubations corresponds to the fragmentation pattern assigned by Han et al. (2013) to the OTA-acyl-glucuronide [10]. Although an additional minor signal was recorded for one of the mass transitions characteristic for glucuronic acid conjugates of OTA (*m/z* 578.0 to 402.0), this was too low to allow firm conclusions regarding potential formation of the phenol- and amino-glucuronides suggested by Han et al. (2013) [10]. Importantly, however, our in vitro data reveal clear species differences in the extent of OTA glucuronidation by liver S9, with significantly more (5–10 times as judged by the signal intensities) OTA-glucuronide being formed in rat and human samples as compared to rabbit and minipig samples.

Despite the relatively high doses of OTA employed in our 2-week study, attempts to unambiguously confirm the presence of OTA-glucuronides in rat urine by direct analysis failed. This is, however, in line with previous analyses of human urine spiked with the phenolic ochratoxin A-8-β-glucuronide, which suggest a much lower sensitivity of LC–MS/MS-based methods for the detection of OTA-glucuronides as compared to the parent compound due to a markedly lower ionization efficiency of the conjugates [8]. To establish the correlation between external dose and OTA-derived glucuronides, we therefore employed an indirect method using enzymatic hydrolysis of the urine samples prior to LC–MS/MS analysis to quantify OTA-derived glucuronides in the urine of OTA-exposed rats. This analysis demonstrates a linear relationship between urinary glucuronide excretion and OTA exposure—a key prerequisite for a biomarker of exposure to serve as a basis for deriving reliable daily intake estimates. Excretion of OTA-derived glucuronides in male rat urine was lower as compared to OTA and accounted for less than 2% of the orally administered dose. However, considering the well-documented sex differences in OTA toxicokinetics [6], it is possible that the rate of OTA-glucuronide excretion in the urine of female rats may differ from that in males. The low excretion rate observed in this study contrasts data obtained from human urine samples, which show up to 6-fold higher concentrations of OTA following enzymatic hydrolysis as compared to those without hydrolysis [8,11], which indicate that, in humans, urinary levels of OTA-glucuronides exceed those of the parent compound. While this may suggest species differences in the extent of OTA glucuronidation in vivo, the currently available human data also indicate considerable inter-individual differences in OTA metabolism and the excretion of OTA-derived phase II conjugates [8,12].

In contrast to OTA-glucuronides, we found no evidence for the formation of putative OTA-derived mercapturic acids to support their use as biomarkers of exposure. Neither OTHQ-NAC, suggested to be formed via cytochrome P450 or peroxidase-mediated oxidation of OTA to a quinone/hydroquinone redox couple (OTQ/OTHQ) and subsequent conjugation with GSH [4,13], nor OTB-NAC, recently reported to be present in some human urine samples at concentrations similar to OTA [9], were detected in rat urine, despite the considerably higher doses of OTA in the animal studies (up to 2 mg/kg bw/day) as compared to human dietary exposure to OTA, even in highly exposed consumers (2.40 to 51.69 ng/kg bw per day). While it is possible that the sensitivity of our method may limit detection of minor metabolites, it should be noted that the method was sufficiently sensitive to detect hydroxy-OTA and OP-OTA, which are also minor metabolites of OTA. Moreover, a signal corresponding to one of the mass transitions characteristic for OTB-NAC (*m/z* 528.9 to 400.1) was detected, yet spiking with OTB-NAC generated by photosynthesis did not confirm the presence of OTB-NAC. Similarly, our in vitro studies on OTA biotransformation using liver S9 from rats, rabbits, minipigs and humans do not suggest that OTB-NAC or the corresponding GSH derivative are formed. In contrast, time-dependent formation of hydroxy-OTA and OP-OTA was consistently observed, with the rates of formation decreasing in the order of minipig > rat > human > rabbit, as judged by the signal intensities.

Surprisingly, a signal corresponding to OTHQ-GSH, first synthesized by Dai et al. (2002) [13], was observed in incubations of OTA with liver S9 in the absence (but not in the presence) of an NADPH regenerating system. The only plausible explanation for this observation may be that, in the presence of NADPH, the quinone (OTQ), which has been postulated to be formed from OTA by oxidation mediated by peroxidases or cytochrome P450 [4], is rapidly reduced to the hydroquinone (OTHQ) via either NAD(P)H:Quinone Oxidoreductase 1, NADPH P450 reductase or possibly P450s (personal communication, F. Peter Guengerich, Vanderbilt University School of Medicine). Since it is likely the quinone (OTQ) that reacts with GSH, whereas the hydroquinone (OTHQ) is unreactive, rapid reduction of the quinone to OTHQ may prevent formation of OTHQ-GSH in the presence of NADPH. From the present and previous work on OTA metabolism in vitro or in vivo [4,13], including studies using [^3^H]-OTA [14,15], it is, however, safe to conclude that under physiological conditions relevant to the situation in vivo, OTHQ-GSH and consequently its corresponding mercapturic acid are not formed at relevant rates that would enable their use as biomarkers of exposure.

## 4. Conclusions

Taken together, results from this study contribute to the establishment of OTA-derived glucuronides as valid biomarkers of OTA exposure by (a) providing direct evidence for glucuronidation of OTA in vitro, particularly in rats and humans, and by (b) demonstrating a linear relationship between urinary excretion of OTA-derived glucuronides and external dose as a prerequisite for deriving probable intakes. Moreover, our in vitro and in vivo data do not support putative OTA metabolites derived from conjugation of OTA with GSH and subsequent processing via the mercapturic acid pathway as biomarkers of exposure.

## 5. Materials and Methods

### 5.1. Chemicals and Reagents

Ochratoxin A (CAS No. 303-47-9; 99% purity), glucose-6-phosphate, glucose-6-phosphate-dehydrogenase (*S. cerevisiae*) and uridine-diphosphate-glucuronic acid trisodium salt were acquired from Sigma Aldrich (Taufkirchen, Germany). U-(^13^C_20_)-ochratoxin A was purchased from Romer Labs GmbH (Butzbach, Germany). Unless otherwise indicated, all other chemicals were purchased from Merck (Darmstadt, Germany), Sigma Aldrich (Taufkirchen, Germany) or Roth (Karlsruhe, Germany).

Liver S9 fractions from rats (Sprague-Dawley, male, 97 donors, 20 mg/mL) and humans (mixed genders, 50 donors, 20 mg/mL) were obtained from XenoTech (Kansas City, USA). S9 fractions from Goettingen^®^ minipigs and rabbits were prepared in-house based on a modified protocol from Hubbard et al. (1985) [16]. Rabbit liver (Blue Vienna x German Giant, female) was obtained from ImmonuGlobe (Himmelstadt, Germany), whereas minipig liver (Goettingen^®^ minipig, male, 42.4 kg bw) was obtained from the University Goettingen, Department of Animal Science.

### 5.2. Photosynthesis of OTA-Derived Glutathione and N-Acetyl-Cysteine Conjugates

OTA conjugates with *N*-acetyl-cysteine (NAC) and glutathione (GSH) were synthesized via photoreaction as previously described [9]. Briefly, reaction solutions containing 20 μg (0.05 μmol, 1.00 eq.) OTA and 123 μg (0.75 μmol, 15.0 eq.) *N*-acetyl-cysteine or 231 μg (0.75 μmol, 15.0 eq.) glutathione in a total volume of 100 µL 0.05 M phosphate-buffered saline (pH = 10) were irradiated for 5, 10, 15 or 20 min in a Rayonet photoreactor (Southern New England Ultraviolet Company; Branford, USA) equipped with a RPR-3500A lamp at a wavelength of 350 nm. Of each reaction solution, 10 µL was directly injected into the LC–MS system for analysis.

### 5.3. In Vitro Incubation of OTA with Liver S9

For analysis of OTA-derived GSH and NAC conjugates, liver S9 fractions (4 mg/mL) of rats, rabbits, minipigs and humans were incubated with OTA (0.1 mM), GSH or NAC (5 mM) and an NADPH regenerating system (NADP (5 mM), glucose-6-phosphate (5 mM), glucose-6-phosphate-dehydrogenase (1.25 units/mL)) in 100 mM phosphate-buffered saline (pH 7.4) in a total volume of 300 µL at 37 °C for two hours. Control incubations were carried out a) in the absence of GSH/NAC and b) in the absence of the NADPH regenerating system. Aliquots (100 µL) were removed immediately (*t* = 0) as well as after 60 (*t* = 60) and 120 (*t* = 120) minutes, and the enzymatic reaction was stopped by addition of 200 µL ACN and centrifugation (20 min, 4 °C, 20,000× *g*). The supernatants were removed and stored at −80 °C until further analysis.

For analysis of OTA-derived glucuronides, liver S9 fractions (4 mg/mL) of rats, rabbit, minipigs and humans were incubated with OTA (0.1 mM), uridine-diphosphate-glucuronic acid (5 mM) and magnesium chloride (5 mM) in 100 mM phosphate-buffered saline (pH 7.4) in a total volume of 300 µL at 37 °C for two hours. Control incubations were carried out in the absence of uridine-diphosphate-glucuronic acid. Aliquots (100 µL) were removed immediately (*t =* 0) as well as after 60 (*t =* 60) and 120 (*t =* 120) minutes, and the enzymatic reaction was stopped by addition of 200 µL ACN and centrifugation (20 min, 4 °C, 20,000× *g*). The supernatants were removed and stored at −80 °C until further analysis. Ten microliters (10 µL) of each sample was injected into the LC–MS system and analyzed using the method described below.

### 5.4. Rat Urine Samples

Archived urine samples from previous repeated dose oral toxicity studies on OTA in male F344/N rats were used [17,18]. The studies were performed according to national animal welfare regulations after authorization by the local authorities (Regierung von Unterfranken, AZ 621-2531.01-9/98 and AZ 54-2531.01-59/05).

14-day study: Male F344 rats (170–190 g) were purchased from Charles-River Laboratories, Sulzfeld, Germany. Animals had free access to water and standard diet (Altromin) and were kept under standard conditions (12 h day/night cycle, temperature 21–23 °C, humidity 45–55%). Rats were transferred to metabolic cages (Tecniplast Metabolic Cage—Rats Over 300 g; Tecniplast, Italy) for collection of urine 3 days prior to dosing. Animals were administered OTA dissolved in corn oil by oral gavage at 0, 0.25, 0.5, 1.0 and 2.0 mg/kg bw for 2 weeks (5 days per week) [17]. Urine was collected every 12 h on day 1 of treatment and every 24 h thereafter on ice, aliquoted and stored at −20 °C until further analysis.

90-day study: Male F344/N rats (6–7 weeks old) were purchased from Harlan-Winkelmann, Borchen, Germany. Animals were housed in groups of 5 in Macrolon type-4 cages and allowed free access to pelleted standard rat maintenance diet (SSNIFF, Soest, Germany) and tap water. After a week of acclimatization, rats (*n* = 5/dose) were exposed to OTA dissolved in corn oil by oral gavage at doses of 0, 21, 70 or 210 µg/kg bw for 2, 4 or 13 weeks (5 days per week). For urine collection, animals were transferred into metabolic cages (Tecniplast Metabolic Cage—Rats Over 300 g; Tecniplast, Italy) 48 h prior to necropsy and fasted for 20 h but allowed free access to tap water. Urine was collected for 24 h on ice, aliquoted and stored at −20 °C until further analysis.

### 5.5. Direct Analysis of OTA Metabolites in Rat Urine

Urine samples were diluted with acetonitrile (2:1), vortexed and cleared via centrifugation (4 °C, 15 min., 2000× *g*) to precipitate proteins. Of the resulting supernatant, 10 µL were injected into the LC–MS system and analyzed using the method described below.

### 5.6. Indirect Analysis of OTA-Derived Glucuronides in Rat Urine

Duplicate analysis of OTA in urine samples with and without enzymatic hydrolysis allowed for indirect measurement of glucuronides, whereby an increase in the concentration of OTA in the samples incubated with enzyme compared to those without enzymatic treatment reflected the hydrolyzed glucuronides. Aliquots of rat urine (125 µL) were mixed with 0.1 M sodium acetate (104 µL; pH = 5). For analysis of the concentration of OTA without enzymatic hydrolysis, an aliquot (30 µL) of this solution was removed and mixed with 7 µL acetonitrile. The remaining solution (199 µL) was incubated with β-glucuronidase (1.65 µL; <100,000 units/mL) at 37 °C for 1 h. Hydrolysis was terminated by addition of ice-cold acetonitrile (50 µL) and centrifugation (15 min., 4 °C, 20,000× *g*). To 98 µL supernatant, 2 µL isotopically labeled ^13^C_20_-OTA in acetonitrile (14-day study: 5 µg/mL; 90-day study: 1 µg/mL) was added as an internal standard. For analysis, 10 µL of each sample were injected into the LC–MS system and analyzed using the method described below. A calibration curve was prepared from solutions of OTA and the isotopically labeled ^13^C_20_-OTA internal standard in acetonitrile and subsequent dilution with urine of a control animal.

### 5.7. LC–MS/MS Analyses

LC–MS/MS analyses were performed on an Agilent 1100 series LC coupled to an API 2000/Q-Trap mass spectrometer (Applied Biosystems/MDS Sciex, Concord, Canada). Samples were injected into the LC–MS/MS system through an Agilent 1100 series autosampler. Separations were carried out on a ReproSil-Pur C18 AQ column (2 mm × 150 mm, 5 µm; Dr. Maisch; Ammerbuch, Germany). Gradient elution of OTA-derived metabolites was carried out with water + 0.1% formic acid (solvent A) and acetonitrile (solvent B). Initially, solvent A was held isocratic for 5 min at 100%, followed by a linear gradient to 95% B in 5 min. These conditions were held for further 5 min. Within 1 min, the gradient decreased linearly to 0% B and remained at initial conditions until the end of analysis (30 min). A flow rate of 0.2 mL/min was used. For each run, 10 µL of the respective sample were injected by the autosampler.

The API 2000/Q-Trap mass spectrometer was operated with a Turbo Ion Spray source in negative ion mode with a voltage of −4000 V. Spectral data were recorded with N_2_ as the heater gas at 450 °C and as the collision gas (CAD = −2) in multiple reaction monitoring mode (MRM). The OTA-derived metabolites and *m*/*z* transitions monitored are summarized in Table 3.

In addition to MRM, constant neutral loss of 129 amu in the negative ion mode based on the characteristic fragmentation pattern of mercapturic acids was used to screen for *N*-acetyl-cysteine conjugates of OTA. Furthermore, constant neutral loss of 176 amu in positive ion mode based on the fragmentation pattern of glucuronic acid conjugates was used to screen for OTA glucuronides [13].

Enhanced product ion spectra for *m*/*z* 545 (OTHQ-NAC), *m/z* 528.9 (OTB-NAC) and *m*/*z* 578 (OTA-Glucuronide) were recorded over the range of *m*/*z* 100–650, *m/z* 100–570 amu and *m*/*z* 100–700, respectively, on an API 2000/Q-Trap mass spectrometer operating in negative ion mode. The collision gas was N_2_ at CAD = −2, and the collision energy was −30 V.

Method validation parameters including apparent recovery, limit of detection (LOD) and quantification (LOQ) were determined according to European Commission Regulation 657/2002/EC [19] by spiking blank urine samples in triplicate with OTA reference standard and ^13^C_20_-OTA as internal standard. Matrix-matched calibration curves were constructed based on the ratio of the peak area of OTA to that of ^13^C_20_-OTA. The mean apparent recovery of OTA from the matrix was calculated by dividing the established concentration by the theoretical concentration, giving a mean value of 89%. The LOD and LOQ of OTA were determined by serial dilution of spiked urine samples and also evaluated by using the signal-to-noise (S/N) ratio of 3:1 and 10:1, respectively. The LOD for OTA in urine was 0.59 ng/mL while the LOQ was 1.97 ng/mL. Calibration was linear in the range of 5 ng/mL to 500 ng/mL.

## Figures and Tables

**Table 1:** Urinary excretion of OTA and OTA-glucuronides expressed in nmol/24 h and fraction of the administered dose (%) following oral treatment of male F344/N rats with OTA at 2 mg/kg bw for 2 weeks (5 days/week). Due to the limited availability of urine samples from this study, data from two individual animals are shown.

Analyte	Animal	Urinary Excretion	Days of Treatment (2 mg OTA/kg bw, 5 d/Week)					
			3	5	7	9	11	13
**OTA**	2	nmol/24 h (%)	15.3 (1.8)	40.3 (4.7)	35.4 (4.1)	32.9 (3.7)	40.9 (4.6)	21.0 (2.2)
	3	nmol/24 h (%)	4.4 (0.5)	30.9 (3.5)	62.4 (6.9)	17.8 (2.0)	33.6 (3.7)	48.1 (5.3)
**OTA-Glucuronide**	2	nmol/24 h (%)	0.6 (0.1)	7.0 (0.8)	0.7 (0.1)	12.2 (1.4)	3.1 (0.4)	3.4 (0.4)
	3	nmol/24 h (%)	1.5 (0.2)	1.6 (0.3)	22.0 (2.4)	16.9 (1.9)	22.5 (2.5)	6.384 (0.7)

**Table 2:** Urinary excretion of OTA and OTA-glucuronides expressed in nmol/24 h and fraction of the administered dose (%) following oral treatment of male F344/N rats with OTA for 2, 4 or 13 weeks (5 days/week). Data are presented as mean ± standard deviation (*n* = 2–5 animals per group).

Analyte	Treatment (Weeks)	Urinary Excretion	OTA Dose (μg/kg bw Per Day)		
			21	70	210
**OTA**	2	nmol/24 h (%)	0.22 ± 0.03 (1.9)	0.32 ± 0.12 (0.8)	1.35 ± 0.36 (1.2)
	4	nmol/24 h (%)	1.88 ± 0.03 (1.5)	0.51 ± 0.09 (0.9)	5.86 * (4.6) *
	13	nmol/24 h (%)	0.18 ± 0.07 (1.1)	0.97 ± 0.36 (1.9)	3.88 ± 1.50 (2.5)
**OTA-Glucuronide**	2	nmol/24 h (%)	0.10 ± 0.02 (0.8)	0.23 ± 0.08 (0.6)	0.48 ± 0.36 (0.4)
	4	nmol/24 h (%)	0.08 ± 0.09 (0.7)	0.61 ± 0.67 (1.5)	0.21 * (0.2) *
	13	nmol/24 h (%)	0.03 ± 0.00 (0.2)	0.85 ± 0.74 (1.7)	1.72 ± 1.53 (1.1)

* *n* = 1.

**Table 3:** HPLC–ESI–MS/MS parameters for potential OTA metabolites.

Analyte	Q1 (*m/z*)	Q3 (*m/z*)	Collision Energy (V)
OTA	402.1	358.0	−31
	402.1	314.0	−47
OTB	368.0	324.0	−30
	368.0	133.0	−30
OTα	255.0	211.0	−40
	255.0	167.0	−40
OH-OTA	418.0	374.0	−30
OP-OTA	420.0	376.0	−30
4-OH-OTB	384.0	340.0	−30
	384.0	192.0	−30
OTHQ	384.0	340.0	−30
	384.0	192.0	−30
OTB-NAC	528.9	400.1	−25
	528.9	312.0	−40
	528.9	208.0	−40
OTHQ-GSH	689.0	560.0	−40
	689.0	416.0	−45
OTHQ-NAC	545.0	416.0	−40
OTB-GSH	673.0	544.0	−30
	673.0	400.0	−30
	673.0	356.0	−30
OTA-glucuronide	578.0	402.0	−24
	578.0	358.0	−13
	578.0	340.0	−30
	578.0	314.0	−30

## Data Availability

Data are contained within the article and Supplementary Material. Raw data are available on request from the corresponding author.

## Supplementary Materials

The following are available online at https://www.mdpi.com/article/10.3390/toxins13080587/s1. Figure S1: LC–MS chromatograms showing analysis of time-dependent formation of potential OTA metabolites in in vitro incubations of OTA with rat liver S9 in the presence of *N*-acetyl-cysteine (**a**) or GSH (**b**), including control experiments carried out in the absence of an NADPH regenerating system. Figure S2: LC–MS chromatograms showing analysis of time-dependent formation of potential OTA metabolites in in vitro incubations of OTA with rabbit liver S9 in the presence of *N*-acetyl-cysteine (**a**) or GSH (**b**), including control experiments carried out in the absence of an NADPH regenerating system. Figure S3: LC–MS chromatograms showing analysis of time-dependent formation of potential OTA metabolites in in vitro incubations of OTA with minipig liver S9 in the presence of *N*-acetyl-cysteine (**a**) or GSH (**b**), including control experiments carried out in the absence of an NADPH regenerating system. Figure S4: LC–MS chromatograms showing analysis of time-dependent formation of potential OTA metabolites in in vitro incubations of OTA with human liver S9 in the presence of *N*-acetyl-cysteine (**a**) or GSH (**b**), including control experiments carried out in the absence of an NADPH regenerating system.
