# Supplementary material for: In Vitro and In Vivo Analysis of Ochratoxin A-Derived Glucuronides and Mercapturic Acids as Biomarkers of Exposure
Source: Toxins (Basel). 2021 Aug 23;13(8):587. doi: 10.3390/toxins13080587 (PMC8402547; doi:10.3390/toxins13080587)
Supplement: Supplementary file 1 [file toxins-13-00587-s001.zip › toxins-1336459-supplementary.pdf]

toxins

MDPI

Supplementary Materials: In Vitro and In Vivo Analysis of Ochratoxin A-Derived Glucuronides and Mercapturic Acids as Biomarkers of Exposure

Raphael Dekant, Michael Langer, Maria Lupp, Cynthia Adaku Chilaka and Angela Mally

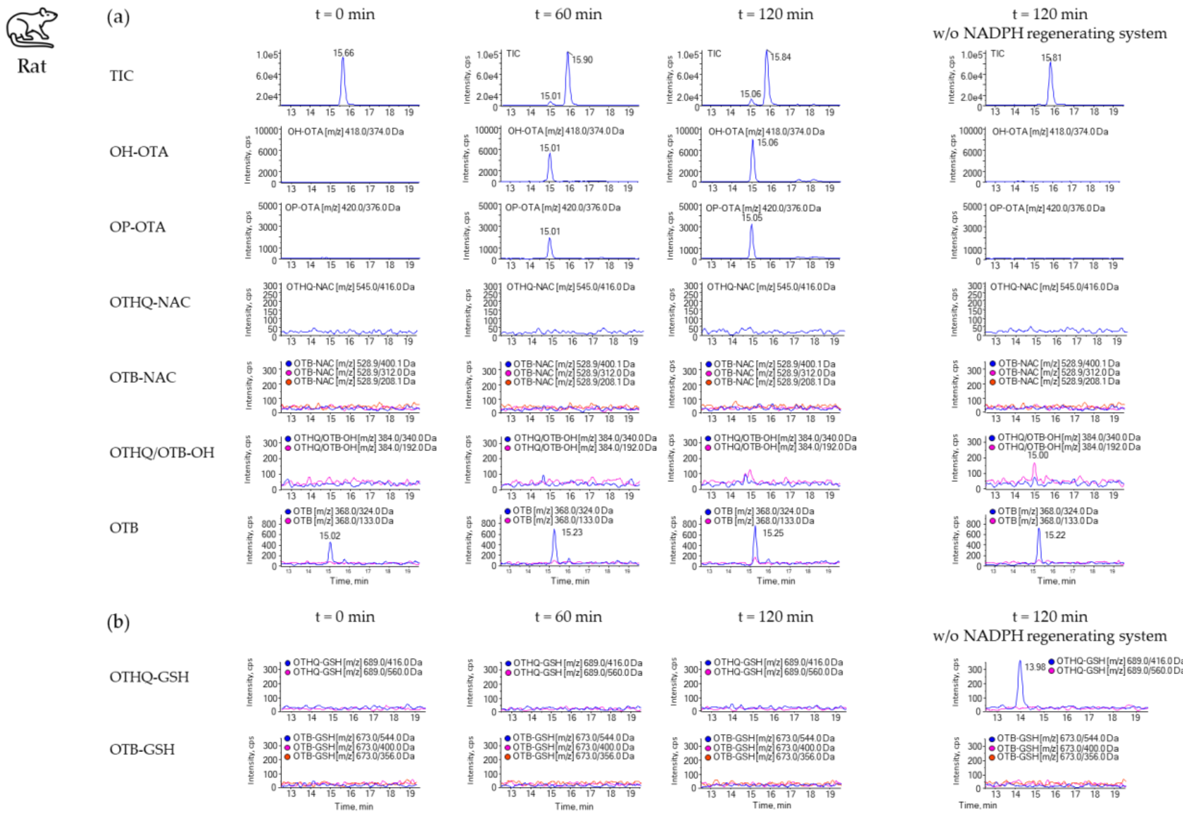

Figure S1. LC-MS chromatograms showing analysis of time-dependent formation of potential OTA metabolites in in vitro incubations of OTA with rat liver S9 in the presence of *N*-acetyl-cysteine (**a**) or GSH (**b**), including control experiments carried out in the absence of an NADPH regenerating system.

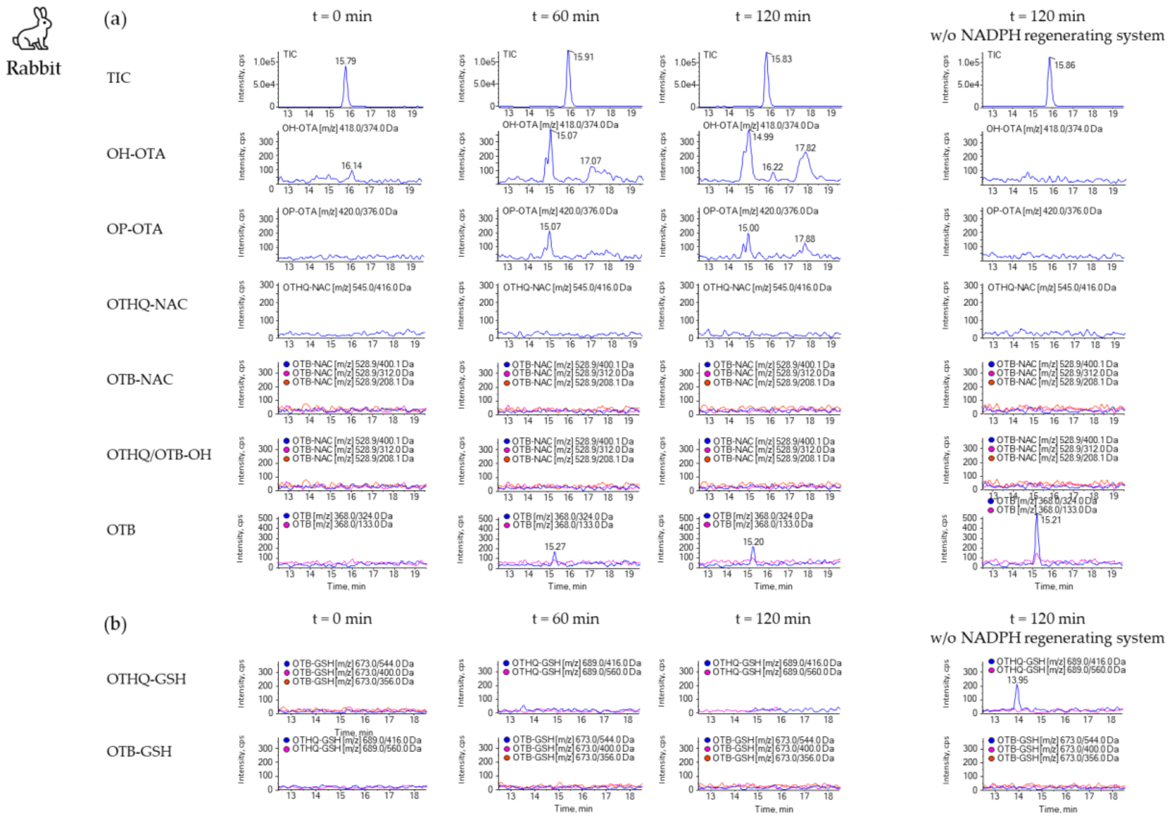

Figure S2. LC-MS chromatograms showing analysis of time-dependent formation of potential OTA metabolites in in vitro incubations of OTA with rabbit liver S9 in the presence of *N*-acetyl-cysteine (**a**) or GSH (**b**), including control experiments carried out in the absence of an NADPH regenerating system.

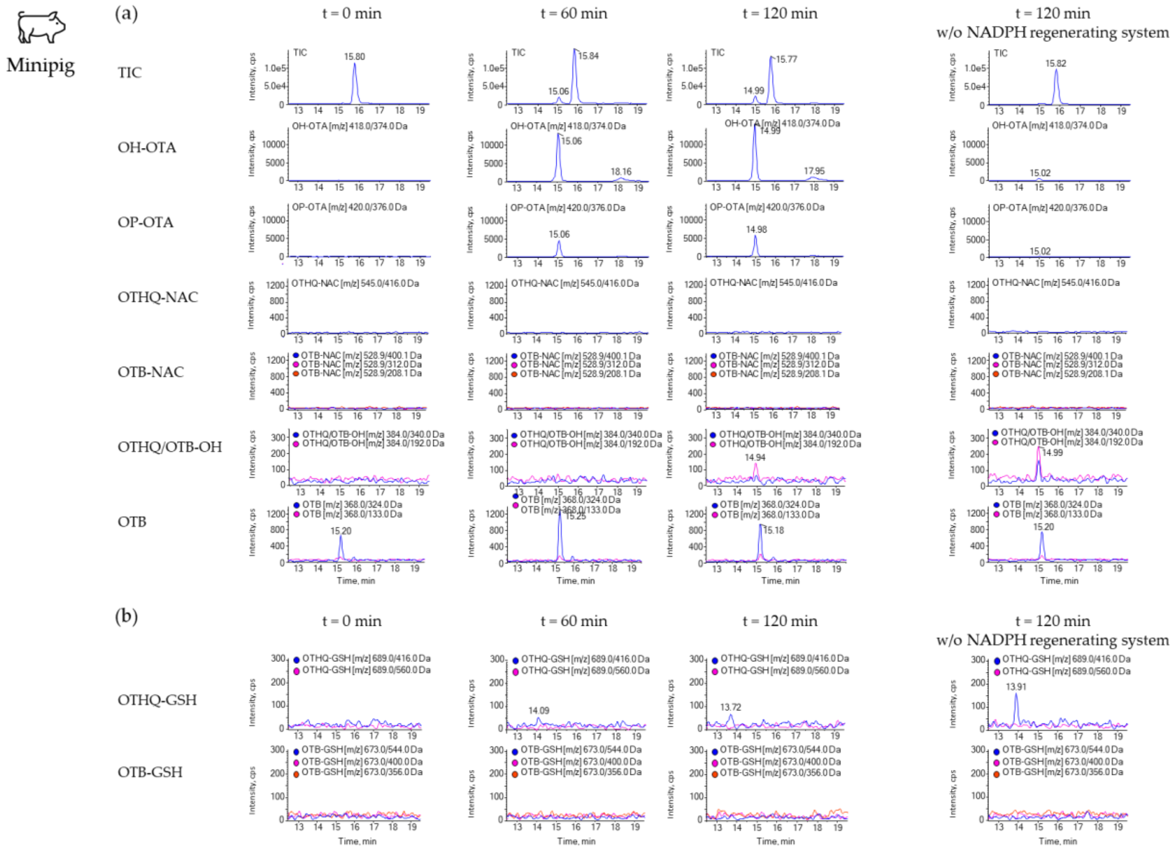

Figure S3. LC-MS chromatograms showing analysis of time-dependent formation of potential OTA metabolites in in vitro incubations of OTA with minipig liver S9 in the presence of *N*-acetyl-cysteine (**a**) or GSH (**b**), including control experiments carried out in the absence of an NADPH regenerating system.

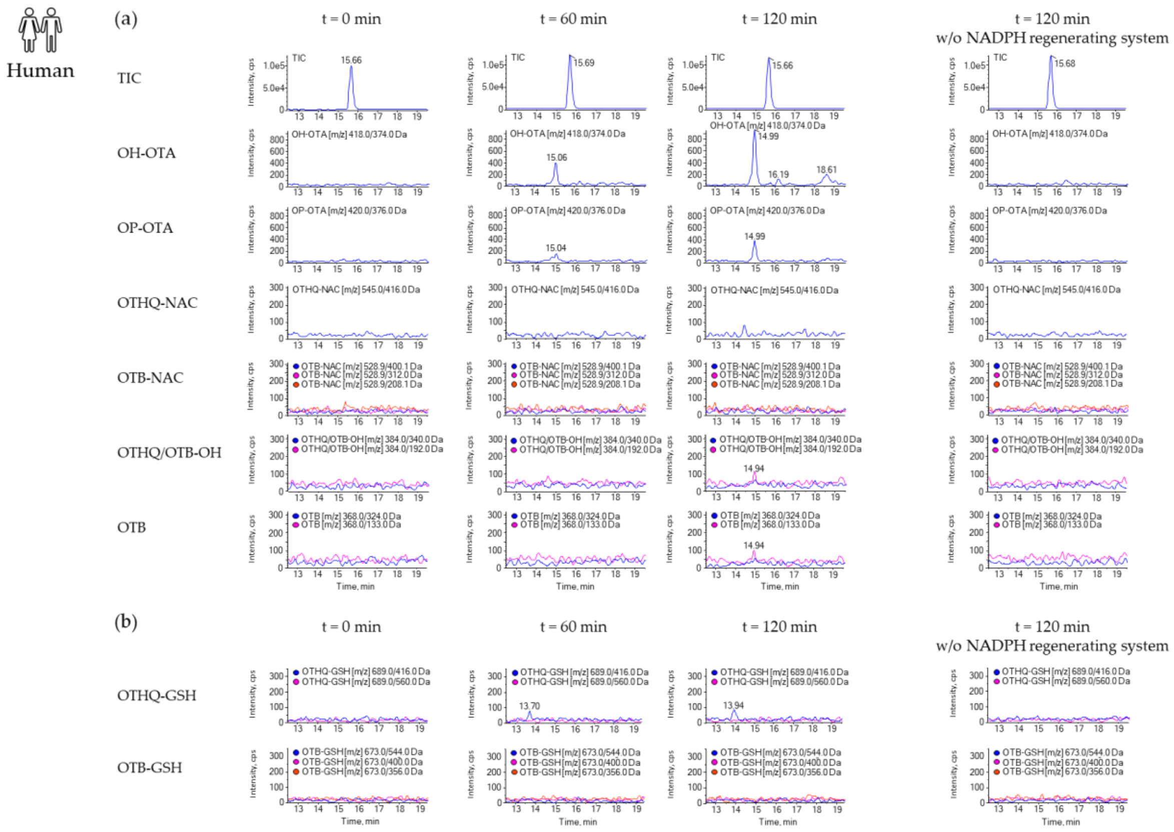

Figure S4. LC-MS chromatograms showing analysis of time-dependent formation of potential OTA metabolites in in vitro incubations of OTA with human liver S9 in the presence of *N*-acetyl-cysteine (**a**) or GSH (**b**), including control experiments carried out in the absence of an NADPH regenerating system.
